# Oral Toxicity and Intestinal Transport Mechanism of Colloidal Gold Nanoparticle-Treated Red Ginseng

**DOI:** 10.3390/nano6110208

**Published:** 2016-11-11

**Authors:** Song-Hwa Bae, Jin Yu, Mi-Ran Go, Hyun-Jin Kim, Yun-Gu Hwang, Soo-Jin Choi

**Affiliations:** 1 Division of Applied Food System, Major of Food Science and Technology, Seoul Women’s University, Seoul 01797, Korea; songhwa29@naver.com (S.-H.B.); ky5031@swu.ac.kr (J.Y.); miran8190@naver.com (M.-R.G.); kimhj043@naver.com (H.-J.K.); 2 SMNANOBIO Co., Ltd., Daejeon 34028, Korea; ykhwang80@gmail.com

**Keywords:** gold nanoparticle, red ginseng, oral toxicity, transport mechanism

## Abstract

(1) Background: Application of nanotechnology or nanomaterials in agricultural food crops has attracted increasing attention with regard to improving crop production, quality, and nutrient utilization. Gold nanoparticles (Au-NPs) have been reported to enhance seed yield, germination rate, and anti-oxidant potential in food crops, raising concerns about their toxicity potential. In this study, we evaluated the oral toxicity of red ginseng exposed to colloidal Au-NPs during cultivation (G-red ginseng) in rats and their intestinal transport mechanism. (2) Methods: 14-day repeated oral administration of G-red ginseng extract to rats was performed, and body weight, hematological, serum biochemical, and histopathological values were analyzed. An in vitro model of human intestinal follicle-associated epithelium (FAE) and an intestinal epithelial monolayer system were used for intestinal transport mechanistic study. (3) Results: No remarkable oral toxicity of G-red ginseng extract in rats was found, and Au-NPs did not accumulate in any organ, although Au-NP transfer to G-red ginseng and some increased saponin levels were confirmed. Au-NPs were transcytozed by microfold (M) cells, but not by a paracellular pathway in the intestinal epithelium. (4) Conclusion: These findings suggest great potential of Au-NPs for agricultural food crops at safe levels. Further study is required to elucidate the functional effects of Au-NPs on ginseng and long-term toxicity.

## 1. Introduction

Nanotechnology and nanomaterials have attracted much attention in diverse fields, including analysis, catalytic process, biosensors, cosmetics, pharmaceutics, medicine, food industry, and agriculture [1,2]. Recently, application of nanotechnology or nanoparticles (NPs) in agricultural processes and products has become an emerging area [3,4]. Nanotechnology can improve crop production, crop quality, nutrient utilization, plant growth and yield, and plant disease resistance [5,6]. For example, nanomaterials can be utilized as fertilizers or pesticides, and applied for nano-encapsulated formulation or agri-food production [7,8]. Many researchers have focused on the application of metal-based (MB) or carbon-based (CB) NPs to agriculture; they have demonstrated improved plant growth and development in spinach, peanut, and tomato by titanium dioxide (TiO_2_), iron oxide (Fe_2_O_3_), and multiwalled carbon nanotubes (MWCNTs) [9,10,11]. These positive changes seem to be attributed to enhanced photosynthetic activity and nitrogen metabolism [12,13]. The effects of a fullerene derivative, fullerol (C_60_(OH)_20_), on enhanced plant biomass, fruit yield, and anticancer phytomedicine content in bitter melon have been also demonstrated [3]. Moreover, mechanistic studies on gene expression profiles have revealed that TiO_2_ and MWCNT could induce positive changes in photosynthetic carbon reaction in spinach and growth enhancement in tobacco cells, respectively [4,9,13,14].

In the meantime, there are growing concerns about the accumulation of NPs in food crops and their potential undesirable effects on biological systems. The uptake, translocation, and accumulation of NPs, such as metal oxide, metallic, and CB-NPs, into edible plant cells have been reported in several studies [5,8,15], although some conflicting results have been obtained [16,17]. Furthermore, little is currently known about the potential adverse effects of NPs on agricultural food crops, food chain, environment, and human body. Effects of MWCNT, aluminum, alumina, zinc, and zinc oxide NPs on seed germination and root growth of edible crop plants (radish, rape, ryegrass, lettuce, corn and cucumber) have been evaluated, showing different toxicity depending on the tested NP type [18]. Growth inhibition and bioaccumulation of copper NPs in the terrestrial plants mung bean (*Phaseolus radiatus*) and wheat (*Triticum aestivum*) have been also reported [19].

Few attempts have been made to apply gold (Au)-NPs in agricultural food crops. Nevertheless, Au-NPs have been found to possess positive effects on germination index in cucumber and lettuce [20]. Enhanced seed yield, seed germination rate, vegetative growth, and free radical scavenging activity in *Arabidopsis thaliana* exposed to Au-NPs have been also demonstrated [21]. Recent research has shown that Au-NP fertilizer can enhance the anti-inflammatory effects of red ginseng on mouse peritoneal macrophages [22]. Moreover, Au is generally considered a low toxic material, and no significant oral toxicity or low systemic absorption of colloidal Au-NPs in rats have been reported [23].

Our previous research has demonstrated that colloidal Au-NPs exhibited no cytotoxicity in human intestinal cells up to 13 μg/mL after 24 h exposure, and no acute oral toxicity was observed after 14-day repeated administration to rats, up to 1300 μg/kg, suggesting their low in vitro and in vivo toxicity [23]. The aim of this study was to further evaluate oral toxicity of red ginseng exposed to Au-NPs (G-red ginseng) during cultivation in rats. Moreover, the intestinal transport mechanism of colloidal Au-NPs was investigated using an in vitro model of human intestinal follicle-associated epithelium (FAE) and an intestinal epithelial monolayer system.

## 2. Results

### 2.1. Characterization

Colloidal Au-NPs produced by an electrolysis method were in the size range of 5 to 15 nm and had zeta potential values of −3.37 ± 0.28 mV, as determined by our previous research [23]. The stability of colloidal Au-NPs was determined to be more than 60 days. The Au-NPs were then treated during the cultivation process of six-year-old ginseng for 6 weeks (three times per two weeks). Saponin analysis revealed that Au-NP-treated ginseng had higher Rg1, Re, Rf, Rb1 and Rd levels than untreated ginseng controls (Table 1). In addition, total saponin contents significantly increased by 3.5-fold in Au-NP-treated ginseng. Thusly obtained Au-NP-treated ginseng was then used to obtain G-red ginseng after a steam treatment and drying process. After the steaming process, only the levels of Rg3 (0.892 ± 0.020 mg/kg) and Rd (0.210 ± 0.014 mg/kg) in G-red ginseng extract significantly increased compared to untreated red ginseng extract controls (0.652 ± 0.056 and 0.115 ± 0.01 mg/kg for Rg3 and Rd, respectively) [22].

In order to confirm Au-NP transfer to G-red ginseng, inductively coupled plasma-mass spectrometry (ICP-MS) analysis was performed. G-red ginseng and G-red ginseng extract (60 Brix) were determined to have 0.763 and 0.104 ppm Au content, respectively, which were not evidently found in untreated red ginseng controls.

### 2.2. Body and Organ Weights, Food Intake and Water Consumption

**Figure 1:**
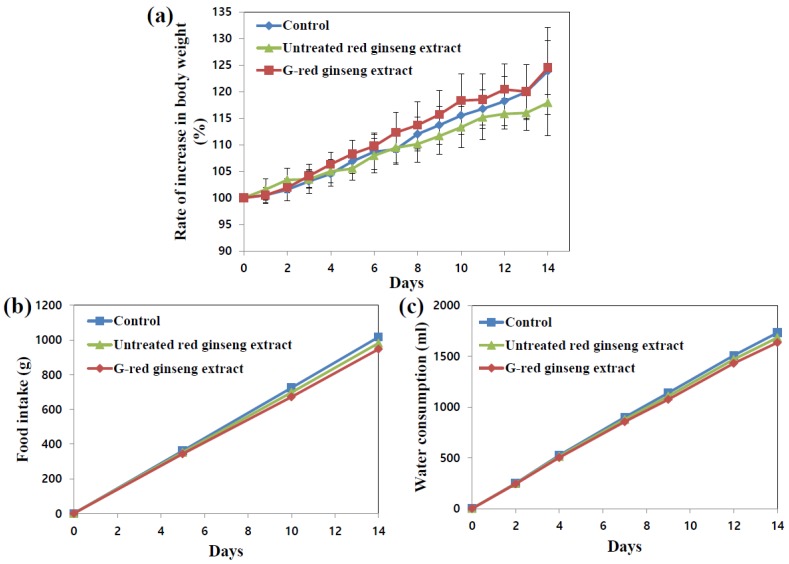
Changes in (**a**) body weight gain; (**b**) food intake; and (**c**) water consumption in rats administered G-red ginseng extract, untreated red ginseng extract, or distilled water (DW) as a control.

In vivo toxicity of G-red ginseng extract was evaluated after 14-day repeated oral administration to rats. G-red ginseng extract of 25 Brix was used for animal experiment, because administration of higher concentration via oral gavage was not possible due to its high viscosity. As shown in Figure 1, no significant abnormality in rats administered G-red ginseng extract was found in terms of changes in body weight gain, food intake and water consumption. Evidently, untreated red ginseng extract exhibited no toxicity. When we compared organo-somatic indices (Table 2), representing the proportional sizes of target organs, all organ weights treated with either G-red ginseng extract or untreated red ginseng extract did not significantly change compared to untreated control (rats administered distilled water (DW) as a control).

### 2.3. Hematology, Coagulation Analysis and Serum Biochemistry

Oral toxicity of G-red ginseng extract was further assessed by hematological and biochemical analysis. Table 3 shows that all blood hematological and coagulation time values were not affected by G-red ginseng extract treatment in rats. Moreover, serum biochemistry results revealed that the levels of all serum biochemical parameters in rats administered G-red ginseng extract were not statistically different from those of the controls (DW) or untreated red ginseng extract (Table 4). The experiments were performed on separate days, so two different control groups were used to analyze statistical significance.

### 2.4. Histopathology

**Figure 2:**
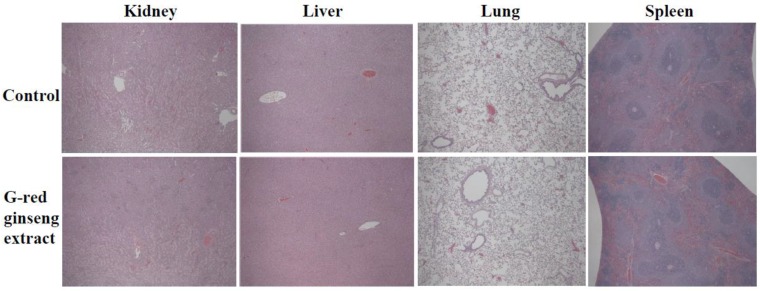
Normal histopathological sections of kidneys, liver, lungs and spleen in the control (DW) and G-red ginseng extract-treated rats after 14-day repeated oral administration. Images are magnified at 50×.

Histopathological examination was also performed to confirm the toxicological effect of G-red ginseng extract on rats. No significant abnormal or pathological changes were observed in kidneys, liver, lungs and spleen in rats treated with G-red ginseng extract (Figure 2). It is worth noting that untreated red ginseng extract caused no pathological abnormality (data not shown).

### 2.5. Tissue Distribution

The possibility of Au-NP accumulation in tissues was assessed in blood, kidneys, liver, lungs and spleen after repeated oral administration to rats for 14 consecutive days. However, Au levels were not detected in all organs analyzed (data not shown).

### 2.6. Intestinal Transport Mechanism

**Figure 3:**
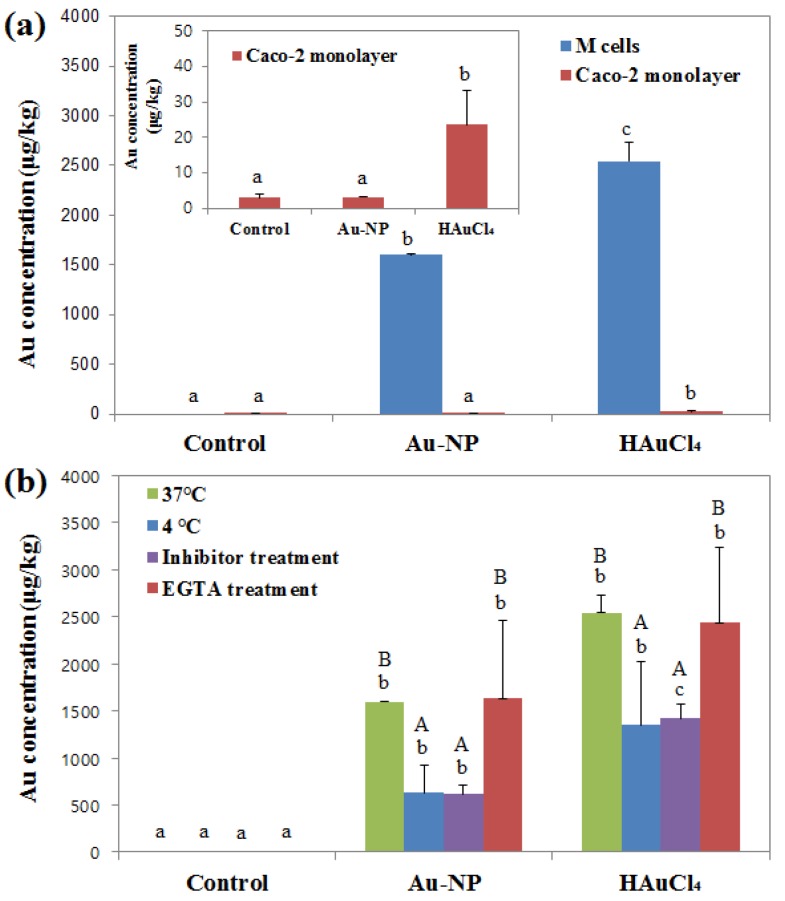
(**a**) Intestinal transport of Au-NPs or Au^3+^ ions using an in vitro follicle-associated epithelium (FAE) model and Caco-2 monolayers; (**b**) Transport amounts of Au-NPs or Au^3+^ ions at different temperatures (37 °C and 4 °C), after pre-treatment with a clathrin-mediated endocytosis inhibitor, chlorpromazine, and after ethylene glycol tetraacetic acid (EGTA) treatment. ^a−c^ Different letters in bars indicate significant differences among control (medium), Au-NP-, and Au^3+^ ion-treated groups (*p* < 0.05). ^A,B^ Different letters in bars indicate significant difference in uptake amounts at 37 °C and 4 °C, with/without inhibitor treatment, and with/without EGTA treatment (*p* < 0.05).

The intestinal transport mechanism of colloidal Au-NPs by microfold (M) cells was evaluated using an in vitro FAE model of co-culture system with intestinal epithelial Caco-2 cells and Raji B lymphocytes. M cells are highly specialized epithelial cells found in the gut-associated lymphoid tissue of the Peyer’s patches and in the mucosa-associated lymphoid tissues, forming part of the FAE. M cells are involved in mucosal immunity and the transport of various molecules including bacteria, viruses, macromolecules, and particles [24]. Figure 3a shows that both Au-NPs and Au^3+^ ions were effectively transported by M cells, but significantly increased Au^3+^ ion transport compared to Au-NPs was found. For comparative study, NP transport was evaluated using Caco-2 monoculture system, which forms a polarized monolayer of enterocytes expressing a dense network of tight junctions, microvilli [25]. Microvilli play a role in the transport of amino acids, bile acid and carboxylic acid, and thus, Caco-2 monoculture is generally used to study the transport of macromolecules [26]. Figure 3a demonstrates that only Au^3+^ ions were found to be transported through Caco-2 monolayers.

NP transport mechanism was further investigated by incubating the in vitro FAE model at 4 °C and comparing the transport amounts between 4 °C and 37 °C to examine the role of the energy-dependent uptake mechanism. The result shows that the translocations of both Au-NPs and Au^3+^ ions significantly decreased at 4 °C as compared with those at 37 °C (Figure 3b).

The role of clathrin-mediated endocytosis in the NP transport was also examined by pre-incubating the cells with chlorpromazine, a clathrin-dependent pathway inhibitor, followed by NP treatment, since M cells are known to be capable of clathrin-mediated endocytosis [27,28]. Figure 3b shows that Au translocation significantly decreased in both Au-NP- and Au^3+^ ions-treated cells after pre-blocking clathrin-mediated endocytosis pathway.

To answer the question as to whether Au-NP transport is mediated by a transcellular or paracellular route, pre-treatment of the FAE model with ethylene glycol tetraacetic acid (EGTA) was carried out, which is known to increase molecule uptake by paracellular pathway [29]. The transported levels of Au-NP and Au^3+^ ions were not affected by EGTA treatment (Figure 3b).

## 3. Discussion

The application potential of colloidal Au-NPs in agricultural food crops was investigated in the present study, applying them during ginseng cultivation as fertilizers, and G-red ginseng were then produced after steam treatment and drying process. ICP-MS results clearly revealed Au-NP transfer to G-red ginseng (0.763 ppm) as well as G-red ginseng extract (0.104 ppm, 60 Brix). Pore diameter of the plant cell wall is known to range from 5 to 20 nm [30], and therefore, NPs with diameter less than 20 nm could easily and effectively pass through the cell membrane [31,32]. The size of Au-NPs used in the present study was determined to be 5–15 nm [23], assuming NP transfer to G-red ginseng. On the other hand, total saponin levels significantly increased (about 3.5-fold) in ginseng exposed to Au-NPs, suggesting that Au-NPs were transferred to ginseng and consequently increased some saponin contents as well as total saponin levels. However, only Rg3 and Rd levels were found to be elevated in G-red ginseng extract compared to untreated red ginseng extract control after the steaming process [22], meaning further optimization of the steam treatment and drying process is needed. It is important to note that saponins are major bioactive components for ginseng functionality, such as lowering blood sugar and cholesterol levels, increase in cognitive function, energy boost, anti-inflammatory effects, cancer prevention, and etc. [33,34]. Hence, these findings suggest great potential of Au-NP treatment for enhanced functionality of red ginseng. Indeed, enhanced anti-inflammatory effects of red ginseng treated with Au-NPs (the same materials used in this study) on macrophages have been recently reported [22]. The positive or negative effects of Au-NPs on ginseng and their functional mechanism are required to be elucidated in the near future.

Repeated oral toxicity study of G-red ginseng extract for 14 consecutive days in rats demonstrated that G-red ginseng extract did not cause any toxicity, as evidenced by no significant changes in body weight gain, behaviors, organo-somatic indices, hematology, serum biochemistry, and histopathology. G-red ginseng extract of more than 25 Brix was found to have too high viscosity, which was not suitable for oral administration. However, this concentration may be high enough to evaluate real oral intake of red ginseng in human body. Indeed, commercially available pure red ginseng extract had about 7 Brix, which was much less than our G-red ginseng extract (25 Brix). Further mechanistic study to determine main the components or interaction between Au-NPs and G-red ginseng components, which contribute functionality or potential toxicity of G-red ginseng, is necessary. On the other hand, Au-NPs did not accumulate in blood or organs after 14-day repeated administration to rats, although increased Au levels (0.104 ppm) were detected in G-red ginseng extract. Our previous study has demonstrated no remarkable oral toxicity of colloidal Au-NPs, the same NPs used in the present study, up to 1300 μg/kg following 14-day repeated oral administration to rats [23]. Low oral absorption (1.85% ± 0.18%) of Au-NPs and their slight accumulation in kidneys have been also found after oral administration of high dose (up to 1300 μg/kg) [23], indicating an extremely low amount entering the bloodstream and possible urinary excretion, which may explain the present findings. Taken together, Au-NPs in G-red ginseng extract were not absorbed into the body, which is probably due to low levels of Au-NPs in G-red ginseng extract and their low oral absorption efficiency. All the results suggest little toxicity potential of G-red ginseng. The toxicity of NPs is highly dependent on material type, and therefore it is important to note that the safety of each material should be determined before food and agricultural application.

Further mechanistic study on the intestinal transport of Au-NPs clearly showed the efficient translocation of Au-NPs by M cells, but not by intestinal epithelial tight junction barrier. Their energy-dependent and transcellular transport by M cells was also confirmed by decrease in transport amounts at 4 °C (61%) and after pre-treatment with clathrin-mediated endocytosis inhibitor (61%). No significantly different transport of Au-NPs after EGTA treatment, which induces the opening of enterocyte tight junctions [29], indicates their transcytosis mechanism by M cells, not by a paracellular route. On the other hand, Au^3+^ ions were found to have different transport mechanism compared to Au-NPs, showing much high transport by M cells and significantly increased translocation through Caco-2 monolayers as well. These results suggest that the intestinal transport mechanism of Au-NPs is primarily governed by M cells, while Au^3+^ ions can be effectively and more massively transported through both M cells and the tight junctions. This can explain higher oral absorption of Au^3+^ ions (8.54% ± 0.65%) than Au-NPs (1.85% ± 0.8%) after a single-dose administration to rats [23]. Moreover, a different biological fate for Au-NPs than that of Au^3+^ ions under physiological conditions could be observed, strongly suggesting the particulate fate of Au-NPs. Understanding in vivo biological fate is of importance to predict the potential toxicity of NPs [35]. It is likely that Au-NPs can be transported across the intestinal epithelium by M cells in particulate forms and absorbed into the bloodstream, but with much less levels than Au^3+^ ions. Therefore, the present results suggest the great potential of Au-NP application in agricultural food crops at safe levels. Further research is required to determine the potential toxicity of Au-NPs after a long-term exposure.

## 4. Materials and Methods

### 4.1. Materials and Characterization

Colloidal Au-NPs (5–15 nm), synthesized by an electrolysis method as described previously [23], were provided from SMNANOBIO Co., Ltd. (Daejeon, Korea) and G-red ginseng extract was obtained from the same company. The prepared Au-NPs were treated during the cultivation process of ginseng for 6 weeks (three times/two weeks). Then, Au-NP-treated ginseng were washed, steamed at 96 °C for 3 h, and dried 60 °C for 24 h followed by 40 °C for 2 weeks to obtain G-red ginseng. The G-red ginseng was extracted with deionized water at 96 °C for 24 h, filtered with filter paper, and concentrated at 60 °C to obtain 60 Brix levels. The Au contents in G-red ginseng and G-red ginseng extract (60 Brix) were analyzed by ICP-MS (ELAN 6100, Perkin-Elmer SCIEX, Norwalk, CT, USA). Saponin compounds in G-red ginseng were analyzed by high performance liquid chromatography (HPLC) according to the methods described by Kang et al. [22].

### 4.2. Animals

Five-week-old female Sprague Dawley (SD) rats weighing 130–150 g were purchased from Nara Biotech Co., Ltd. (Seoul, Korea). Animals were housed in plastic laboratory animal cages in a ventilated room maintained at 20 ± 2 °C and 60% ± 10% relative humidity under a 12 h light/dark cycle. Water and commercial laboratory complete food were provided *ad libitum*. Animals were allowed to acclimate to the environment for 7 days before treatment. All animal experiments were performed in compliance with the guideline issued by the Animal and Ethics Review Committee of Seoul Women’s University.

### 4.3. Oral Toxicity and Tissue Distribution

Five female rats per group were daily administered G-red ginseng extract (25 Brix), ginseng extract without Au-NP treatment, or equivalent volume of DW as a control by oral gavage for 14 consecutive days. Changes in body weight, behaviors, specific symptoms, and food or water consumption were daily recorded after treatment. At the end of experiment, animals were sacrificed by CO_2_ euthanasia and organs were collected. Organo-somatic index was calculated by the following formula: (Weight of the organ (g)/Total body weight (g)) × 100.

Blood samples were collected from the posterior vena cava for hematological and serum biochemical analysis, as described previously [23]. Autohematoanalyzer (ADVIA120E, Bayer, New York, NY, USA), coagulometer (ALC 7000, Instrumentation Laboratory, Orangeburg, IL, USA), and biochemical analyzer (TBA-120FR, Toshiba, Otawara, Japan) were used for hematological, aggregation time, and biochemical analysis, respectively. Histopathological examination was performed on kidneys, liver, lungs, and spleen fixed with 10% neutral buffered formalin and stained with hematoxylin and eosin.

To determine biodistribution of Au-NPs, additional female rats (*n* = 5) were orally administered daily for 14 consecutive days, and samples of blood, kidneys, liver, lungs, and spleen were collected at the end of the experiment (14th day).

### 4.4. Au Quantification in Biolgical Matrices

Au analysis in biological samples was performed as described previously [23]; biological samples (0.2 g of blood or 1 g of tissues) were pre-digested in 20 mL of ultrapure nitric acid and heated at ~180 °C, followed by addition of aqua regia solution (HCl:HNO_3_ = 3:1), and the reaction was continued until the solution was colorless and clear. When the remaining solution was 2 mL, the solution was transferred to another vial, and deionized distilled water was added to a total mass of 8 g. Total Au concentrations were analyzed by ICP-MS (ELAN 6100, Perkin-Elmer SCIEX, Norwalk, CT, USA).

### 4.5. Intestinal Transport Mechanism

#### 4.5.1. 3D Cell Culture for FAE Model

Human intestinal epithelial Caco-2 cells and non-adherent human Burkitt’s lymphoma Raji B cells were purchased from the Korean Cell Line Bank (Seoul, Korea) and grown in Dulbecco’s Modified Eagle Medium (DMEM) and RPMI 1640 medium, respectively, supplemented with 10% fetal bovine serum, 1% non-essential amino acids, 1% l-glutamine, 100 units/mL penicillin, and 100 μg/mL streptomycin at 37 °C in 5% CO_2_ atmosphere.

An in vitro model of human FAE was prepared according to the method described by des Rieux et al. [24] to study NP transport by M cells; Transwell^®^ inserts (SPL LIfescience, Gyeonggi-do, Korea) were coated with Matrigel™ matrix (Becton Dickinson, Bedford, MA, USA) for 1 h, supernatants were removed, and then inserts were washed with DMEM. Caco-2 cells (1 × 10^6^ cells/well) were grown on upper insert sides and incubated for 14 days. Raji B cells (1 × 10^6^ cells/well) in DMEM were then added to basolateral insert compartments, and these co-cultures were maintained for 5 days. Apical medium of cell monolayers was then replaced with a particle suspension containing colloidal Au-NP (13 μg/mL) or an equivalent amount of HAuCl_4_ on the basis of Au content, and treated for 6 h. The concentrations of transported particles in basolateral solutions were determined by measuring total Au levels as described in “Au quantification in biological matrices”.

To investigate the role of energy-dependence mechanism in NP transport, the same experiments were performed at 4 °C and 37 °C. In addition, transcellular NP transport by M cells was evaluated by incubating inserts apically and basolaterally with 2.5 mM EGTA in Han’s Balanced Salt Solution (HBSS, pH 7.4) twice for 15 min at 37 °C. Fresh EGTA was then replaced basolaterally and NP suspensions were added to apical sides of cell monolayers, and incubation was continued for 6 h. Basolateral solutions were sampled and analyzed by ICP-MS. This experiment was repeated three times on three separate days.

#### 4.5.2. 3D Cell Culture for Intestinal Epithelial Monolayers

The monoculture system of Caco-2 cells, representing the intestinal epithelium monolayer of tight junctions, was prepared as follow: Caco-2 cells (4.5 × 10^5^ cells/well) were grown on upper insert sides in the same manner as described in the FAE model, cultured for 21 days, and treated with Au-NPs or an equivalent amount of HAuCl_4_ in the same manner as described in “3D cell culture for FAE model”.

### 4.6. Statistical Analysis

Results were expressed as means ± standard deviations. One-way analysis of variance (ANOVA) with Tukey’s Test in SAS Ver.11.0 (SAS Institute Inc., Cary, NC, USA) was used to determine the significances of intergroup differences. Statistical significance was accepted for *p* values of <0.05.

## 5. Conclusions

Oral toxicity of red ginseng exposed to Au-NPs (G-red ginseng) was evaluated following 14-day repeated administration to rats. No significant toxicity of G-red ginseng extract was found in terms of body weight change, hematology, serum biochemistry, histopathological values, or tissue accumulation, although Au-NP transfer to G-red ginseng and some increased saponin levels were found. On the other hand, Au-NPs were determined to be transcytozed by M cells, but not by a paracellular route in the intestinal epithelium. These findings suggest the great potential of Au-NPs for application in agricultural food crops at safe levels. Further research is required to determine the functional effects of Au-NP treatment on ginseng growth and its potential toxicity after long-term exposure.

## Figures and Tables

**Table 1:** Saponin contents in Au-NP-treated ginseng or untreated ginseng controls.

Type of Ginseng	Rg1	Re	Rf	Rg2	Rb1	Rb3	Rd	Total
Untreated ginseng	1.67	1.43	0.33	0.13	2.48	0.18	0.12	6.34
Au-NP-treated ginseng	14.42	2.93	0.91	0.15	3.42	0.15	1.33	22.31

Concentration: mg/kg.

**Table 2:** Organo-somatic indices of rats after 14-day repeated oral administration of G-red ginseng extract or untreated red ginseng extract.

Organ	Control	Untreated Red Ginseng Extract	G-red Ginseng Extract
Brain	0.92 ± 0.07	1.03 ± 0.13	0.91 ± 0.11
Heart	0.38 ± 0.04	0.37 ± 0.04	0.37 ± 0.04
Kidney	0.97 ± 0.08	1.01 ± 0.11	0.89 ± 0.10
Large Intestine	1.49 ± 0.26	1.51 ± 0.35	1.24 ± 0.29
Liver	4.41 ± 0.20	4.93 ± 0.56	4.12 ± 0.45
Lung	0.52 ± 0.04	0.47 ± 0.18	0.55 ± 0.10
Ovary	0.04 ± 0.01	0.07 ± 0.01	0.05 ± 0.01
Small Intestine	4.27 ± 0.42	4.62 ± 0.04	3.81 ± 0.32
Spleen	0.28 ± 0.02	0.35 ± 0.01	0.28 ± 0.04
Stomach	2.23 ± 0.63	1.60 ± 0.10	1.82 ± 0.30

All data showed no statistical differences from the control (DW) groups (*p* > 0.05).

**Table 3:** Hematological and coagulation time values in rats after 14-day repeated oral administration of G-red ginseng extract or untreated red ginseng extract.

Test Groups	WBC	WBC Differential Counting (%)					RBC	Hb	HCT	MCV	MCH	MCHC	RETI	PLT	PT	APTT
	(10^3^/μL)	NE	LY	MO	EO	BA	(10^6^/μL)	(g/dL)	(%)	(fL)	(pg)	(g/dL)	(%)	(10^3^/μL)	(s)	(s)
**Control 1**	5.4 ± 3.2	12.2 ± 6.9	82.2 ± 7.6	1.7 ± 0.3	1.8 ± 1.3	0.7 ± 0.4	6.5 ± 1.9	12.8 ± 3.4	40.3 ± 11.0	62.4 ± 1.6	20.0 ± 0.7	32.0 ± 0.5	3.0 ± 0.2	515 ± 571	14.0 ± 0.9	41.7 ± 4.4
**Untreated Red Ginseng Extract**	7.8 ± 1.5	7.2 ± 1.6	87.4 ± 2.2	2.1 ± 0.5	1.3 ± 0.2	0.7 ± 0.3	7.7 ± 0.3	15.3 ± 0.5	47.8 ± 1.4	62.5 ± 1.1	19.9 ± 0.3	31.9 ± 0.2	2.4 ± 0.4	1104 ± 305	13.3 ± 0.5	36 ± 8.3
**Control 2**	6.0 ± 0.9	5.8 ± 1.2	88.0 ± 1.4	2.1 ± 0.6	1.5 ± 0.4	0.5 ± 0.3	7.1 ± 0.4	13.7 ± 0.8	47.4 ± 2.6	67.1 ± 1.5	19.4 ± 0.5	28.9 ± 0.2	2.2 ± 0.3	921 ± 44	13.2 ± 0.6	23.2 ± 1.9
**G-red Ginseng Extract**	6.6 ± 1.2	7.1 ± 3.5	86.6 ± 3.6	2.2 ± 0.5	1.8 ± 0.4	0.6 ± 0.1	7.6 ± 0.5	14.4 ± 0.8	49.4 ± 2.6	65.1 ± 1.3	19.0 ± 0.4	29.2 ± 0.3	2.19 ± 0.33	890 ± 173	14.1 ± 0.9	27.4 ± 6.9

Abbreviation: WBC, total leucocyte count; NE, neutrophils; LY, lymphocytes; MO, monocytes; EO, eosinophils; BA, basophils; RBC, total erythrocyte count; Hb, hemoglobin concentration; HCT, hematocrit; MCV, mean cell volume; MCH, mean cell hemoglobin; MCHC, mean cell hemoglobin concentration; RETI, reticulocyte; PLT, platelet; PT, prothrombin time; APTT, activated partial thromboplastin time. Statistical analysis of untreated red ginseng extract and G-red ginseng extract were performed compared to control 1 and control 2, respectively. Data were expressed as means ± standard deviations. All treated groups showed no significant differences from the control 1 or control 2 (distilled water) group (*p* > 0.05).

**Table 4:** Serum biochemical values in rats after 14-day repeated oral administration of G-red ginseng extract or untreated red ginseng extract.

Test Groups	TP	ALB	A/G	T-BIL	ALP	AST	ALT	CREA	BUN	CHOL	TG	GLU	CA	IP	CK	Na	K	Cl
	(g/dL)	(g/dL)	-	(mg/dL)	(U/L)	(U/L)	(U/L)	(mg/dL)	(mg/dL)	(mg/dL)	(mg/dL)	(mg/dL)	(mg/dL)	(mg/dL)	(IU/L)	(mmol/L)	(mmol/L)	(mmol/L)
**Control 1**	6.9 ± 0.2	4.3 ± 0.2	1.7 ± 0.1	0.0 ± 0.0	851 ± 218	85 ± 4	34 ± 5	0.5 ± 0.1	20.9 ± 2.1	96 ± 7	113 ± 16	366 ± 105	12.2 ± 0.8	9.6 ± 0.7	178 ± 49	145.6 ± 1.6	6.6 ± 0.7	99.7 ± 1.8
**Untreated Red Ginseng Extract**	8.8 ± 0.0	5.0 ± 0.1	1.4 ± 0.1	0.0 ± 0.0	864 ± 115	174 ± 21	46 ± 11	0.5 ± 0.1	22.6 ± 2.3	93 ± 14	106 ± 12	461 ± 277	12.9 ± 1.0	11.8 ± 1.3	1735 ± 230	141.5 ± 0.8	11.0 ± 0.5	101.6 ± 2.5
**Control 2**	6.5 ± 0.2	4.3±0.1	2.0 ± 0.0	0.0 ± 0.0	1116 ± 117	76 ± 7	37 ± 5	0.5 ± 0.1	21.4 ± 3.2	83 ± 6	90 ± 19	286 ± 51	13.1 ± 0.2	9.8 ± 0.7	112 ± 10	147.5 ± 1.0	5.5 ± 0.5	97.1 ± 1.2
**G-red Ginseng Extract**	6.7 ± 0.2	4.5±0.1	2.0 ± 0.1	0.0 ± 0.0	904 ± 148	77 ± 7	26 ± 4	0.5 ± 0.1	21.4 ± 2.0	86 ± 8	52 ± 28	268 ± 43	12.8 ± 0.5	9.1 ± 0.9	113 ± 18	148.4 ± 1.5	5.4 ± 0.6	98.2 ± 2.7

Abbreviation: TP, total protein; ALB, albumin; A/G, A/G ratio; T-BIL, total bilirubin; ALP, alkaline phosphatase; AST, aspartate aminotransferase; ALT, alanine aminotransferase; CREA, creatinine; BUN, blood urea nitrogen; CHOL, total cholesterol; TG, triglycerides; GLU, glucose; CA, calcium; IP, inorganic phosphorus; CK, creatine kinase; Na, sodium; K, potassium; Cl, chloride. Statistical analysis of untreated red ginseng extract and G-red ginseng extract were performed compared to control 1 and control 2, respectively. Data were expressed as means ± standard deviations. All treated groups showed no significant differences from the control 1 or control 2 (DW) group (*p* > 0.05).
